# Correction: Control of Arabidopsis shoot stem cell homeostasis by two antagonistic CLE peptide signalling pathways

**DOI:** 10.7554/eLife.112605

**Published:** 2026-07-14

**Authors:** Jenia Schlegel, Gregoire Denay, Rene H Wink, Karine Gustavo Pinto, Yvonne Stahl, Julia Schmid, Patrick Blümke, Rüdiger GW Simon

**Keywords:** *A. thaliana*

Schlegel J, Denay G, Wink R, Pinto KG, Stahl Y, Schmid J, Blümke P, Simon RGW. 2021. Control of Arabidopsis shoot stem cell homeostasis by two antagonistic CLE peptide signalling pathways. *eLife* **10**:e70934. doi: 10.7554/elife.70934.

Published 13 October 2021

We noticed that the reporter line used to investigate the number of WUSCHEL-expressing cells in Figure 7 was unstable and mislabelled as “pWUS:NLS-GFP”. Instead, the reporter line in Col-0 was obtained from Pfeiffer et al. (2016) and carries a pWUS::3xVenus-NLS transgene conferring BASTA resistance. During the introduction of this reporter into the *cle40-2* background, no BASTA selection was applied, the resulting F3 generation was not stable and the pWUS::3xVenus-NLS transgene segregated. Therefore, our observation that *cle40-2* shows reduced pWUS::3xVenus-NLS activity is not based on the analysis of homozygous transgenic plants and we can no longer support this statement.

Consequently, as a precautionary measure, we removed Figure 7, including Figure 7—figure supplement 1 and Figure 7—figure supplement 2 as well as all statements based on these data. The new Figure 7 was previously included as Figure 7—figure supplement 3. Accordingly, we also provide an adjusted model (new Figure 8) for CLE40 function in the inflorescence meristem.

Corrected Figure 8 (changes to legend text underlined):

**Figure 1:**
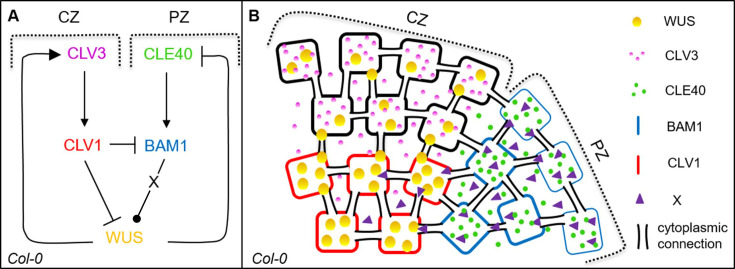


**Schematic model of two intertwined signalling pathways in the shoot meristem.**

(**A, B**) Schematic representation of two intertwined signalling pathways in the inflorescence meristem (IFM) of *Arabidopsis thaliana*. CLV3 in the central zone (CZ) binds to the LRR receptor CLV1 to activate a downstream signalling cascade which leads to the repression of the transcription factor WUS. In a negative feedback loop WUS protein moves to the stem cells to activate *CLV3* gene expression. In the peripheral zone (PZ) of the IFM, a second signalling pathway controls meristem growth by CLE40 and its receptor BAM1. CLE40 binds to BAM1 in an autocrine manner, leading to the activation of a downstream signal ‘X’ which affects WUS activity or expression. WUS protein represses the expression of the *CLE40* gene. Arrows indicate a promoting effect, the blocked line indicates a repressing signal, and lines terminating in a circle indicate that the molecular nature of interaction is unclear.

Originally published Figure 8 shown for reference:

**Figure 2:**
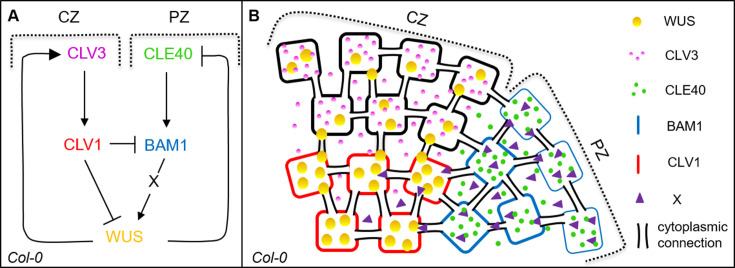


**Schematic model of two intertwined signalling pathways in the shoot meristem.**

(**A, B**) Schematic representation of two negative feedback loops in the inflorescence meristem (IFM) of *Arabidopsis thaliana*. CLV3 in the central zone (CZ) binds to the LRR receptor CLV1 to activate a downstream signalling cascade which leads to the repression of the transcription factor WUS. In a negative feedback loop WUS protein moves to the stem cells to activate *CLV3* gene expression. In the peripheral zone (PZ) of the IFM, a second negative feedback loop controls meristem growth by CLE40 and its receptor BAM1. CLE40 binds to BAM1 in an autocrine manner, leading to the activation of a downstream signal ‘X’ which promotes WUS activity. WUS protein in turn represses the expression of the *CLE40* gene. Arrows indicate a promoting effect, and the blocked line indicates a repressing signal.

The removed figures, Figure 7, Figure 7—figure supplement 1 and Figure 7—figure supplement 2, are shown below for reference.

Removed Figure 7:

**Figure 3:**
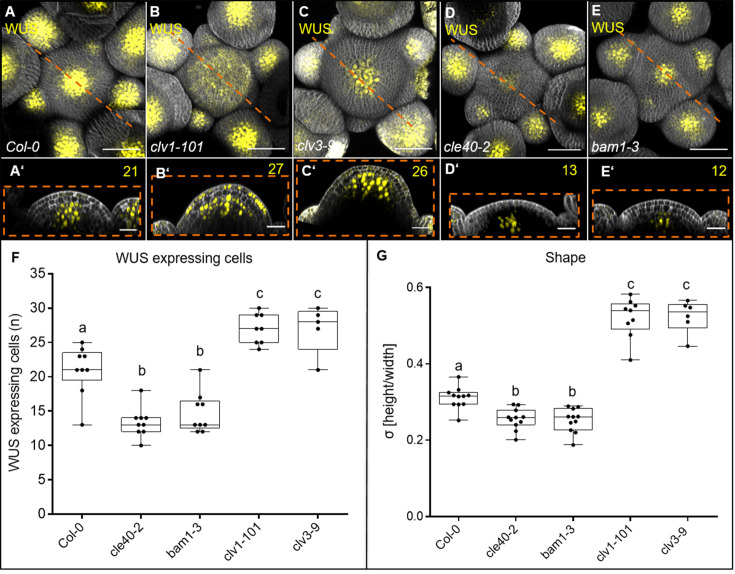


**CLE40 and BAM1 promote** ***WUS*** **expression.**

(**A–E’**) Maximum intensity projection (MIP) and longitudinal optical section of inflorescences at 5 weeks after germination (WAG) expressing the transcriptional reporter *WUS:NLS-GFP* in a (**A, A’**) *Col-0*, (**B, B’**) *clv1-101,* (**C, C’**) *clv3-9*, (**D, D’**) *cle40-2* and (**E, E’**) *bam1-3* background. In (**A**) wild-type plants, the WUS domain is smaller compared to the expanded WUS domain in (**B**) *clv1-101* and (**C**) *clv3-9* mutants. The WUS domain of (**D**) *cle40-2* and (**E**) *bam1-3* mutants is decreased compared to wild-type plants. Longitudinal optical sections of (**B’**) *clv1-101* and (**C’**) *clv3-9* mutants expand along the basal-apical axis while the meristem shape of (**D’**) *cle40-2* and (**E’**) *bam1-3* mutants is flatter compared to (**A’**) wild-type plants,. (**F**) Box and whisker plot shows the number of *WUS*-expressing cells in a single plane through the organizing centre (OC) of inflorescence meristems (IFMs) of *Col-0* (N=9), *cle40-2* (N=9), *bam1-3* (N=9), *clv1-101* (N=8) and *clv3-9* (N=5). (**G**) At 5 WAG, *bam1-3* (N=11) and *cle40-2* (N=11) mutants have flatter meristems than wild-type plants (decreased σ value compared to *Col-0* [N=11]), while *clv1-101* [N=9] and *clv3-9* [N=6] mutants increase in their IFM height showing a higher σ value. Scale bars: 50 µm (**A–E**), 20 µm (**A’–E’**), Statistical groups and stars were assigned after calculating p-values by ANOVA and Tukey’s multiple comparison test (differential grouping from *P*≤0.01). yellow numbers: *WUS*-expressing cells in the CZ; σ value: height/width of IFMs.

Removed Figure 7—figure supplement 1:

**Figure 4:**
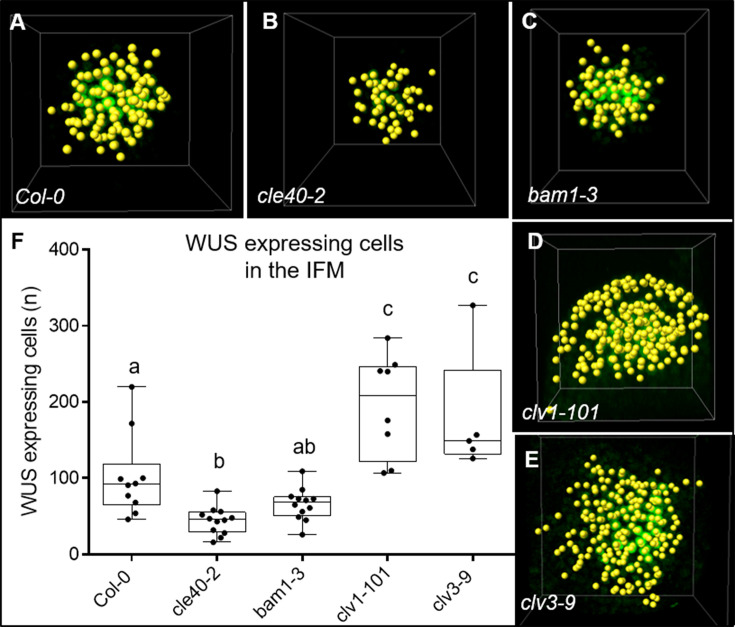


**Number of** ***WUS*****-expressing cells in the inflorescence meristem (IFM) of various mutant backgrounds detected with Imaris software.**

(**A–E**) Spot detection of *WUS*-expressing cells in the IFMs of (**A**) *Col-0*, (**B**) *cle40-*2, (**C**) *bam1-3*, (**D**) *clv1-101* and (**E**) *clv3-9* mutants via Imaris software. (**F**) Box and whisker plot shows the number of *WUS*-expressing cells that were detected by the Imaris software in the IFMs of *Col-0* (N=9), *cle40-2* (N=9), *bam1-3* (N=9), *clv1-101* (N=8) and *clv3-9* (N=5). Statistical groups were assigned after calculating p-values by ANOVA and Tukey’s multiple comparison test (differential grouping from *P*≤0.05).

Removed Figure 7—figure supplement 2:

**Figure 5:**
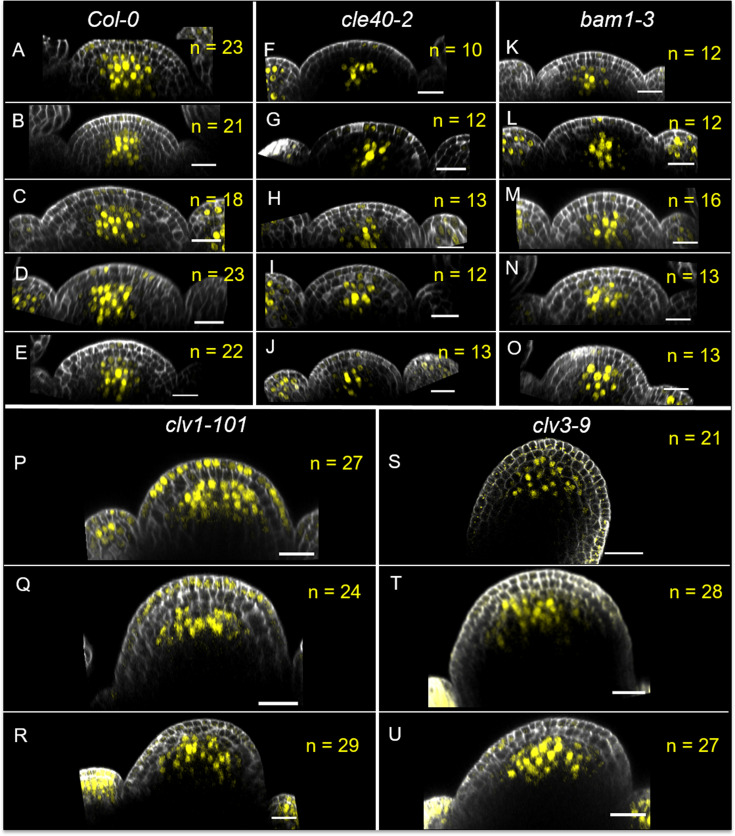


**Number of** ***WUS*****-expressing cells in a longitudinal section through the meristem in multiple inflorescence meristems (IFMs).**

(**A–U**) Longitudinal optical sections through the IFM of 3–5 (**A–E**) *Col-0*, (**F–J**) *cle40-2,* (**K–O**) *bam1-3*, (**P–R**) *clv1-101* and (**S–U**) *clv3-9* plants expressing the transcriptional reporter *WUS:NLS-GFP*. Scale bars: 20 µm (**A–U**), yellow numbers: *WUS*-expressing cells in the central zone (CZ).

